# The Delivery of Extracellular “Danger” Signals to Cytosolic Sensors in Phagocytes

**DOI:** 10.3389/fimmu.2022.944142

**Published:** 2022-07-14

**Authors:** Gerone A. Gonzales, Johnathan Canton

**Affiliations:** ^1^ Department of Biochemistry and Molecular Biology, Cumming School of Medicine, University of Calgary, Calgary, AB, Canada; ^2^ Faculty of Veterinary Medicine, University of Calgary, Calgary, AB, Canada; ^3^ Calvin, Joan and Phoebe Snyder Institute for Chronic Diseases, University of Calgary, Calgary, AB, Canada

**Keywords:** phagocyte, dendritic cell, macrophage, pattern recognition receptor, endocytic organelle, DAMP, PAMP, phagocytosis

## Abstract

Phagocytes, such as macrophages and dendritic cells, possess the ability to ingest large quantities of exogenous material into membrane-bound endocytic organelles such as macropinosomes and phagosomes. Typically, the ingested material, which consists of diverse macromolecules such as proteins and nucleic acids, is delivered to lysosomes where it is digested into smaller molecules like amino acids and nucleosides. These smaller molecules can then be exported out of the lysosomes by transmembrane transporters for incorporation into the cell’s metabolic pathways or for export from the cell. There are, however, exceptional instances when undigested macromolecules escape degradation and are instead delivered across the membrane of endocytic organelles into the cytosol of the phagocyte. For example, double stranded DNA, a damage associated molecular pattern shed by necrotic tumor cells, is endocytosed by phagocytes in the tumor microenvironment and delivered to the cytosol for detection by the cytosolic “danger” sensor cGAS. Other macromolecular “danger” signals including lipopolysaccharide, intact proteins, and peptidoglycans can also be actively transferred from within endocytic organelles to the cytosol. Despite the obvious biological importance of these processes, we know relatively little of how macromolecular “danger” signals are transferred across endocytic organelle membranes for detection by cytosolic sensors. Here we review the emerging evidence for the active cytosolic transfer of diverse macromolecular “danger” signals across endocytic organelle membranes. We will highlight developing trends and discuss the potential molecular mechanisms driving this emerging phenomenon.

## Introduction

Phagocytes serve a central role in the maintenance of organismal homeostasis and immunity. Through their ability to ingest large quantities of extracellular material *via* the specialized endocytic processes of macropinocytosis and phagocytosis, phagocytes can scavenge cellular debris, such as apoptotic cells or material derived from necrotic cells, ingest and kill invading pathogens and survey the environment for signs of potential “danger”. Both phagocytosis and macropinocytosis are active processes and the constitutive ingestion of exogenous material represents a significant metabolic burden. For example, phagocytosis of an apoptotic cell results in the acquisition of macromolecules including proteins, nucleic acids, lipids and carbohydrates (1). As such, phagocytes have in place machinery for the breakdown and recycling of phagocytic and macropinocytic cargo. Phagosomes undergo sequential interactions with endocytic organelles and lysosomes and also acquire v-ATPases which results in the gradual acidification of the lumen and the simultaneous acquisition of degradative enzymes with acidic pH optima. Whereas macropinosomes undergo membrane crenation and shrinkage events that allow for the recycling of membrane back to the plasma membrane as well as for their cargo to be delivered to lysosomes where it can be digested (2, 3). Endocytosed macromolecules are therefore digested into smaller molecules such as amino acids and nucleosides which can be exported to the cytosol *via* transmembrane transporters for incorporation into the phagocyte’s metabolic pathways or for altogether export from the cell *via* plasma membrane transporters (4).

**Figure 1:**
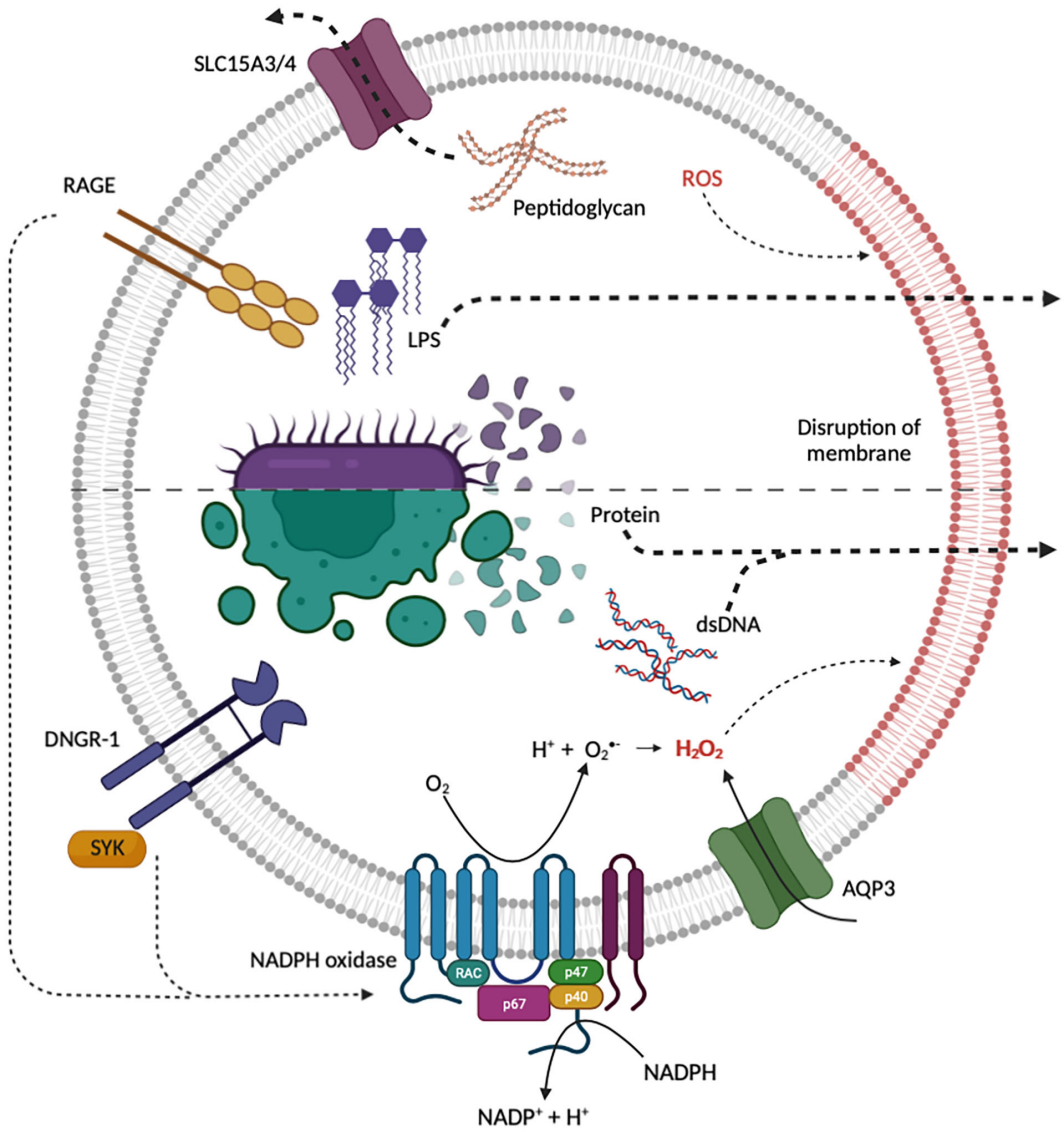
Current proposed mechanisms for the endosomal escape of diverse macromolecular “danger” signals from endocytic organelles. Internalized bacteria can release peptidoglycan, LPS and bacterial protein which may gain access to the cytosol through transporters or induced membrane destabilization. Both RAGE and DNGR-1 induce NADPH oxidase ROS production, triggering ROS mediated membrane damage and the release of protein, dsDNA and LPS. NADPH oxidase and AQP3 may be recruited to endocytic organelles to induce ROS-dependent membrane damage and lipid peroxidation. Red lipids indicate disruption of endosomal membrane by ROS. Created with BioRender.com.

There are, however, exceptions – not all macromolecules ingested by phagocytes are digested in phagosomes and macropinosomes. In some cases, intact macromolecules are exported to the cytosol (**Figure 1**). For example, it has been known for decades that, in dendritic cells, intact endocytosed proteins can be transferred across the phagosomal membrane to the cytosol where they are processed for antigen cross-presentation [reviewed in (5–7)]. Cross-presentation is fundamental to the ability of immune cells to detect potential “danger” in the form of pathogen-derived proteins and neoantigens and to instigate immunity. Organisms deficient in cross-presentation become dangerously susceptible to infection and tumor challenge (5–13). More recently, there is mounting evidence that in addition to proteins, many other macromolecules are actively transported across phagosomal membranes. In the tumor microenvironment, double stranded DNA (dsDNA) in tumor cell debris is phagocytosed by dendritic cells and macrophages and transferred to the cytosol where it activates the cGAS-STING pathway (14–16). Similarly, during bacterial infection, macrophages distal to the site of infection macropinocytose lipopolysaccharide (LPS) and transfer it to the cytosol for inflammatory signalling (17–20). Despite the obvious importance of these processes to the ability of macrophages and dendritic cells to detect “danger” and to instigate immunity, our understanding of how macromolecules are delivered to the cytosol of phagocytes is in its infancy.

Here we review the evidence for the newly emerging concept of phagocyte-concerted transfer of endocytosed macromolecules to the cytosol. We discuss the physiological consequences of and attempt to provide perspectives on potential mechanisms for the cytosolic transfer of endocytosed macromolecules by phagocytes.

## Delivering endocytosed proteins to the cytosol

Some of the earliest evidence for the transfer of endocytosed macromolecules to the cytosol came from the study of antigen cross-presentation. Antigen cross-presentation is the process by which antigen presenting cells, primarily dendritic cells, initiate cytotoxic T lymphocyte responses to many types of viruses and tumors (21–23). There are believed to be two pathways for cross-presentation. In the first, referred to as the vacuolar pathway, exogenous proteins are processed into peptides and loaded onto histocompatibility complex class I (MHC-I) molecules within the lumen of endocytic organelles (24–26). The peptide-MHC-I complexes are then transported to the cell surface where they can serve as a ligand for T cell receptors on CD8 T cells. In the second pathway, known as the cytosolic pathway of antigen cross-presentation, intact or partially degraded proteins are transferred from within endocytic organelles, including phagosomes and macropinosomes, to the cytosol (27–30). Evidence for the transfer of intact or partially degraded proteins come from the finding that enzymes retain their enzymatic activity upon transfer to the cytosol (31–33). Once in the cytosol, the proteins are processed into peptides by the proteasome (27, 34, 35). The peptides are finally loaded onto MHC-I molecules in the endoplasmic reticulum and transported to the cell surface to activate CD8+ T lymphocytes (5). But exactly how proteins escape degradation in endocytic organelles and are then transported across endocytic organelle membranes to the cytosol remains unclear (6).

Although several phagocytes can perform cross-presentation *in vitro*, dendritic cells excel at cross-presentation relative to other cell types and most of the *in vivo* evidence points to dendritic cells as the primary cross-presenting cells (7, 9, 10). It is therefore conceivable that dendritic cells may express specific antigen export machinery that allows for the transfer of endocytosed proteins to the cytosol. This notion is bolstered by several seminal findings. For example, when cytochrome *c* is injected into the blood stream of mice, it results in the selective depletion of a group of cross-presenting dendritic cells known as classical (or sometimes also referred to as conventional) dendritic cells type 1 (cDC1) (32). The implication is that cytochrome c is endocytosed by these cells and subsequently transferred from within endocytic organelles to the cytosol where it induces APAF-1-dependent cell death (32). Importantly, this selective depletion of cDC1s completely ablates *in vivo* cross-presentation. Similarly, in mice lacking the transcription factor BATF3, *in vivo* cross-presentation of protein antigens from virally infected cells and tumor cells is completely lost (10). BATF3 is required for the development of cDC1s and the loss of cross-presentation is likely due to the complete absence of cDC1s, although other BATF3-dependent functions may also contribute. It is also worth noting that very recently another group of spleen-resident phagocytes, known as red pulp macrophages, have also been shown to be capable of cross-presenting model protein antigens *in vivo (* 36). Together, these findings suggest that dendritic cells, particularly cDC1s, and perhaps some types of macrophages are specialized in cross-presentation and may express unique machinery for the transfer of intact proteins across endocytic organelle membranes. But what might this specialized machinery look like?

Several hypotheses exist for the transfer of endocytosed proteins across endocytic organelle membranes. One hypothesis that has been extensively studied is the “transporter hypothesis”. The transporter hypothesis states that the translocation of proteins from within endocytic organelles to the cytosol is mediated by a protein transporter (5, 6). Early evidence from proteomics analyses of phagosomes (37), which was later corroborated by high resolution microscopy (31, 38, 39), found that phagosomes in dendritic cells and macrophages contain components of the endoplasmic reticulum including proteins involved in endoplasmic reticulum-associated degradation (ERAD) (40). This led to a series of studies describing a role for the endoplasmic reticulum-resident trimeric protein channel SEC61 in the translocation of proteins from within endocytic organelles, including phagosomes, to the cytosol. SEC61 is an ERAD protein that is believed to be responsible for the retro-translocation of peptides from the lumen of the endoplasmic reticulum to the cytosol for subsequent degradation (41). Inhibition of SEC61 with the bacterial toxin exotoxin A [ExoA (41)] results in reduced cross-presentation. Similarly, the knockdown of SEC61 with siRNA, as well as the exclusion of SEC61 from endocytic organelles with an ER-retaining intrabody diminishes cross-presentation. Cells transfected with a dominant-negative form of the p97 AAA ATPase, a hexametric protein that interacts with proteins undergoing translocation *via* SEC61, failed to export luciferase from within endocytic organelles to the cytosol and failed to cross-present exogenous antigen (39, 42). Altogether, these findings point to a potential role for the SEC61 translocon in the export of endocytosed proteins from endocytic organelles to the cytosol.

The precise role played by SEC61 in the delivery of endocytosed proteins to the cytosol for cross-presentation is nevertheless difficult to deduce. Indeed, much of the work on SEC61 in cross-presentation has been performed using tools that result in the chronic inhibition or mislocalization of SEC61 or other ERAD proteins. Such chronic inhibition may result in the inhibition of MHC-I-dependent presentation by other off-target effects that are not related to protein export from endocytic organelles. For example, SEC61 inhibition can result in reduced trafficking of MHC-I from the endoplasmic reticulum to the plasma membrane (43). To circumvent this limitation, a very recent study employed a newly described tool – mycolactone – for potent and acute SEC61 inhibition (43). Acute inhibition of SEC61 with mycolactone did reduce cross-presentation, however, contrary to previous findings, it did not correlate with the inhibition of the endosome-to-cytosol export of endocytosed proteins (43). Instead, the inhibition of cross-presentation by SEC61 resulted from the diminished expression of proteins required for cross-presentation, including MHC-I. These findings directly contradict SEC61-mediated protein translocation out of endocytic organelles (43). It is therefore unclear at present what role, if any at all, the ERAD machinery has in the transport of endocytosed proteins to the cytosol.

In the last five years, another model for the transfer of endocytosed proteins to the cytosol has been gaining traction. The “indigestion model” was first described nearly thirty years ago by Reis e Sousa and Germain (28). This model states that destabilization of endocytic organelle membranes leads to rupture and the subsequent release of cargo, including protein antigens, into the cytosol (28). One proposed mechanism of membrane destabilization is through the peroxidation of lipids in the limiting membrane of endocytic organelles by reactive oxygen species (ROS) (**Figure 1**). ROS can modify lipid bilayers through the direct oxidization of polyunsaturated lipid tails. Oxidized lipid tails bend towards the water phase and this conformational change affects the thickness, fluidity, and permeability of the bilayer (44–46). Studies by Dingjan et al. suggest that endosomal ROS produced by the NOX2-containing NADPH-oxidase can cause lipid peroxidation (47, 48). When cells are stimulated with lipopolysaccharide (LPS), a pathogen-associated molecular pattern (PAMP) that is known to promote NADPH oxidase activity, there is an increase in peroxidation of lipids on endocytic organelles resulting in rupture and the leakage of endocytosed proteins to the cytosol (47). Similarly, α-Tocopherol, an antioxidant which scavenges the ROS produced by the NADPH oxidase, reduces ROS-induced lipid peroxidation as well as the release of endocytosed proteins into the cytosol (47). Further evidence in support of ROS-induced membrane destabilization was provided by Nalle et al. who demonstrated that aquaporin-3 (AQP3) transports hydrogen peroxide into endocytic organelles where it contributes to lipid peroxidation, membrane destabilization and protein release to the cytosol (49). It is worth mentioning, that apart from ROS-dependent lipid modification, other lipid modifications on endocytic organelles, such as the accumulation of sphingosine-based lipids, have been proposed although there is no direct evidence for these modifications in the escape of endocytosed proteins into the cytosol as yet [reviewed in (50)].

As ROS-induced destabilization of endocytic organelle membranes may induce cellular toxicity, it is likely that the process is tightly regulated. Evidence for regulation of this sort was recently provided in a study on the dendritic cell receptor DNGR-1 (11, 51). DNGR-1, also known as CLEC9A, is a transmembrane C-type lectin receptor on the surface of cDC1s (12). The ligand for DNGR-1 is filamentous (F-)actin and myosin complexes associated with dead cell debris (11, 12, 52). When DNGR-1 binds to its ligand it recruits and activates the kinase SYK to phagosomes harboring dead cell debris (11). DNGR-1-SYK signalling results in NADPH oxidase-dependent ROS production in phagosomes leading to phagosomal rupture and the efflux of proteins from the lumen of the phagosome to the cytosol (11) (**Figure 1**). Using three-dimensional (3D) corelative light and electron microscopy (CLEM) and serial block face (SBF) scanning electron microscopy (SEM), the rupture of the phagosome was visualized, showing a hole with a diameter between 1-1.5 μm (11). This process is believed to regulate the cross-presentation of dead cell associated proteins by cDC1s (11, 13, 51).

The observation that endocytic organelle rupture is regulated by receptors that sense “danger” suggests that rupture or endocytic organelle “indigestion” is inducible - a likely adaptation to guard against unnecessary or constitutive damage to endocytic organelles. Any toxicity associated with endocytic organelle damage may also be offset by mechanisms of membrane repair such as the endosomal sorting complex required for transport (ESCRT) machinery or galectins both of which mediate the repair of damaged endocytic organelles (53–55). The kinetics and regulation of endocytic organelle membrane destabilization have yet to be fully elucidated.

In summary, phagocytes exert mechanisms for the active transfer of endocytosed proteins to the cytosol. This has primarily been studied in the context of cross-presentation and the most recent evidence points to inducible mechanisms that allow for the nonselective delivery of endocytic organelle cargo to the cytosol. This implies that other endocytosed macromolecules may also be released through these pathways. In the next section, we discuss the evidence for the release of another endocytosed macromolecule - double stranded DNA (dsDNA) - to the cytosol.

## Delivering endocytosed double stranded DNA to the cytosol

Double stranded DNA (dsDNA) is often shed by dead and dying pathogens or damaged cells at sites of tissue damage (**Figure 1**). Immune cells harbor pattern recognition receptors that recognize dsDNA as a “danger” signal. For example, in dendritic cells present in the necrotic core of tumors, the cytosolic dsDNA receptor cyclic GMP-AMP synthase (cGAS) is activated by tumor cell-derived dsDNA which leads to stimulator of interferon genes (STING)-dependent inflammatory signalling (14–16). The activation of dendritic cells in this way is critical – impairment of this pathway in dendritic cells renders organisms dangerously susceptible to tumor challenge (14, 16). But how does dsDNA, a macromolecule shed by dead or dying tumor cells, end up in the cytosol of dendritic cells to activate the cGAS-STING pathway?

Recent evidence suggests that the delivery of dsDNA to the cytosol of dendritic cells is likely a two-step process involving first the internalization of extracellular dsDNA into endocytic organelles and second the escape of dsDNA to the cytosol. In line with this, dsDNA shed by dying cells forms complexes with proteins that serve to both prevent its degradation by extracellular DNase-I and to facilitate its endocytosis by phagocytes. For example, the antimicrobial peptide LL37 (also known as CAMP) facilitates the endocytosis of extracellular dsDNA by dendritic cells (56, 57). The interaction between dsDNA and LL37 converts non-stimulatory self-dsDNA into an effective “danger” signal which stimulates inflammatory signalling in phagocytes including dendritic cells and monocytes (56, 57). In monocytes in particular, LL37-dsDNA complexes escape from endocytic organelles to activate the cGAS-STING pathway. Interestingly, LL37-dsDNA escape into the cytosol is enhanced in the presence of the V-ATPase inhibitor bafilomycin (57). The relationship between V-ATPase activity, which acidifies the lumen of endocytic organelles, and the escape of macromolecules is unclear. It is, however, conceivable that bafilomycin impairs the degradation of endocytosed dsDNA by DNase II, which functions optimally under acidic conditions.

More recently, other proteins have been shown to complex with extracellular dsDNA to facilitate its endocytosis and subsequent release to the cytosol. High mobility group box 1 (HMGB1) and other HMGB proteins function as sentinels for extracellular nucleic acids (58). HMGB1 forms complexes with dsDNA leading to its endocytosis (59, 60). Although TLR2 and TLR4 have been implicated in the endocytosis of HMGB1-dsDNA complexes by phagocytes, the exact mechanism and receptor(s) involved remain unclear. HMGB1-dsDNA uptake does however appear to be regulated by the inhibitory receptor T cell immunoglobulin and mucin domain containing (TIM)-3 in dendritic cells, as TIM-3 blockade results in increased endocytosis of extracellular HMGB1-dsDNA complexes (14). Endocytosis of HMGB1-dsDNA complexes results in the transfer of dsDNA to the cytosol where it activates the cGAS-STING pathway in the dendritic cells (14). HMGB1-dependent dsDNA uptake and cytosolic transfer appears to be particularly important in the context of tumors where necrotic tumor cells shed dsDNA which is taken up in the form of HMGB1-dsDNA complexes. The subsequent triggering of the cGAS-STING pathway in the dendritic cells leads to robust anti-tumor immunity (14, 16).

Finally, dsDNA present in neutrophil extracellular traps (NETs) can also trigger the cytosolic cGAS-STING pathway in macrophages (61, 62). Neutrophils release NETs through a specialized form of cell death known as NETosis. These NETs physically trap and kill microbes in a web-like complex of neutrophil derived antimicrobial peptides, granular proteins, neutrophil elastase and dsDNA (61, 62). After NETosis has occurred, macrophages phagocytose the NETs and NET-derived dsDNA escapes the phagosome into the cytosol to activate cGAS (62). Ultrastructural analysis revealed that NETs interact with cGAS after escaping from phagosomes (62). Although the mechanism of dsDNA escape into the cytosol following the phagocytosis of NETs remains unclear, it appears to involve neutrophil elastase, a protein which is abundant in NETs and that can bind to dsDNA. How neutrophil elastase may promote the phagosomal escape of dsDNA is not yet known (62).

In summary, there is growing evidence that phagocytes can endocytose and subsequently deliver dsDNA to the cytosol for the activation of cGAS. This results in inflammatory signalling and is already emerging as an important feature in the generation of anti-tumor immunity. Nevertheless, how dsDNA, a membrane impermeant macromolecule, can cross endocytic organelle membranes to gain access to cytosolic danger sensors is a mystery. Findings thus far suggest that dsDNA requires complexing with protein chaperones to trigger robust cytosolic recognition and to elicit inflammatory signalling, but there has yet to be any proposed mechanisms on how these chaperones allow dsDNA to gain access to the cytosol. Interestingly, the delivery if dsDNA to the cytosol from within endocytic organelles results in the activation of dendritic cells and the upregulation of costimulatory molecules and cytokines such as type I interferons (15). This in turn promotes the cross-presentation of protein antigens (15). It is tempting to speculate that the mechanism(s) governing the release of protein antigens and the release of dsDNA from endocytic organelles are related and perhaps interconnected. To the best of our knowledge, there are no studies investigating whether NADPH oxidase-dependent ROS production and peroxidation of the limiting membranes of endocytic organelles also regulates the escape of endocytosed dsDNA to the cytosol in phagocytes.

## Other macromolecular “danger” signals that are delivered to the cytosol of phagocytes

In addition to proteins and dsDNA, there is growing evidence that other macromolecular “danger” signals can be endocytosed and subsequently transferred to the cytosol of phagocytes to instigate inflammatory signal. This has mostly been observed in the context of infection. For example, in a bacterial infection, pathogen-derived peptidoglycan, flagellin, and lipopolysaccharide (LPS) are delivered to the cytosol where they are detected by cytosolic pattern recognition receptors (**Figure 1**). In some instances, this is due to pathogen-concerted mechanisms, such as cell penetrating peptides or secretion systems and toxins, that allow for cytosolic invasion. These processes will not be discussed here as it has been recently reviewed elsewhere [see (63–66)]. Instead, we discuss the emerging evidence for the active or phagocyte-concerted transfer of endocytosed PAMPs to the cytosol.

Peptidoglycan is a major component of the cell wall of gram-positive bacteria. It is recognized as a “danger” signal and serves as a ligand for the cytosolic pattern recognition receptors nucleotide-binding oligomerization domain (NOD)-like receptors (NLRs), NOD1 and NOD2 (67). The activation of NOD1 and NOD2 leads to pro-inflammatory signalling *via* the activation of NF-κB and mitogen-activated protein kinase (MAPK) (67). During an infection, peptidoglycan is internalized by phagocytes either by phagocytosis of the pathogenic bacteria or by the macropinocytosis of peptidoglycan derivatives, such as muramyl dipeptide, shed by invading bacteria. As the resultant phagosomes and macropinosomes mature, they acquire V-ATPases which acidify the lumen. The proton gradient generated by acidification facilitates the transport of peptidoglycan derivatives to the cytosol *via* proton-coupled oligopeptide transporters (POT) also known as solute carrier family 15 (SLC15), including SLC15A3 and SLC15A4 - both of which are highly expressed in macrophages and dendritic cells (68–70) (**Figure 1**). Of note, and similar to the cytosolic translocation of proteins and dsDNA described above, internalization of peptidoglycan into an endocytic organelle is a prerequisite for its delivery to the cytosol. This two-step process for the detection of peptidoglycan has recently been used to link the constitutive macropinocytosis of macrophages and dendritic cells to their immune surveillance function (71). Indeed, blockade of constitutive macropinocytosis in primary human macrophages renders them incapable of sensing extracellular peptidoglycan derivatives (71). Whether constitutive macropinocytosis contributes significantly to the delivery of other extracellular “danger” signals is currently not known.

Another extracellular PAMP that is actively delivered to cytosolic pattern recognition receptors is LPS. LPS, also known as endotoxin, is a major component of the cell wall of gram-negative bacteria. LPS is well known to elicit inflammatory signalling that is dependent upon its recognition by the transmembrane pattern recognition receptor TLR4. However, it has recently been demonstrated that endocytosed LPS is also delivered to the cytosol where it can bind and activate caspase-11, which cleaves gasdermin D (GSDMD) (17–20, 72, 73). When activated, GSDMD assembles into a pore on the plasma membrane which cause pyroptosis, a lytic form of cell death (72, 73). But how is LPS actively delivered to cytosolic pattern recognition receptors? Interestingly, the mechanism involved in delivering LPS to the cytosol shares several features with the delivery of dsDNA to the cytosol. During a state of sepsis, HMGB1 released from hepatocytes complexes with extracellular LPS. The HMGB1-LPS complexes bind to receptors for advanced glycation end-products (RAGE) on the surface of macrophages and are subsequently internalized into endocytic organelles and delivered to lysosomes (19). In the acidic environment of lysosomes, HMGB1-LPS complexes destabilize and ultimately permeabilize the limiting membrane of the lysosomes. This leads to the leakage of HMGB1-LPS complexes into the cytosol and to the activation of caspase-11 (19). Although it is currently unclear how HMGB1-LPS complexes result in membrane permeabilization, whole-cell patch clamping revealed an HMGB1-dependent inward current presumably caused by HMGB1-dependent membrane permeabilization (19). In addition to the similarities between the delivery of LPS and dsDNA to the cytosol, it is worth noting that the requirement of engagement of the receptor RAGE for subsequent delivery of LPS to the cytosol shares some similarities with the DNGR-1-dependent endocytic organelle destabilization discussed above. Both RAGE and DNGR-1 trigger robust NADPH oxidase-dependent ROS production (11, 74–76) (**Figure 1**). However, whether ROS production downstream of RAGE engagement contributes to the observed lysosome membrane destabilization that results in LPS release to the cytosol remains to be determined.

Apart from HMGB1-dependent membrane permeabilization other pathways have been described for the delivery of endocytosed LPS to the cytosol. Guanylate binding proteins (GBPs) are interferon-γ-inducible GTPases capable of restricting the replication of bacteria and promoting noncanonical inflammasome activation (77–82). GBPs can bind directly to cytosolic pathogens, but can also be recruited to pathogen-containing phagosomes where they instigate phagosomal rupture (77). Phagosomal rupture leads to the delivery of pathogen-derived LPS to the host cytosol. Once in the cytosol LPS activates caspase-11 and downstream inflammatory signalling (77, 78). Although the lysis of pathogen-containing phagosomes by GBPs is well-documented, the molecular mechanism(s) by which GBPs discern “self” endomembranes from the limiting membranes of pathogen-containing phagosomes remains to be determined. It is tempting to speculate that receptors may survey the luminal contents of the phagosomes for PAMPs or DAMPs and initiate signalling that in some way marks the limiting membrane for detection by GBPs and is a promising avenue for future investigation.

Apart from peptidoglycan and LPS, other pathogen-derived PAMPs which actively delivered to the cytosol of phagocytes are being studied. For example, flagellin, the principal structural component in flagella, is shed by some types of bacteria in structures called outer membrane vesicles (OMVs) (83). The OMVs are subsequently endocytosed by macrophages and the flagellin is delivered to the cytosol where it is detected by the cytosolic sensor neuronal apoptosis inhibitory protein 5 (NAIP5). When bound to flagellin, the flagellin-NAIP5 complex assembles with NOD-like receptor family, caspase activation recruitment domain (CARD) domain-containing protein 4 (NLRC4) to form a pro-inflammatory signalling complex called the inflammasome (83, 84). Although less is known about how flagellin gains access to the cytosol, it is likely to involve an HMGB1-RAGE-dependent pathway as OMVs also contain LPS and have been shown to facilitate the cytosolic delivery of LPS (17).

## Conclusion

The phagocyte-concerted delivery of extracellular, seemingly membrane impermeant, “danger” signals to the cytosol to activate cytosolic pattern recognition receptors is emerging as a fundamental mechanism by which phagocytes carry out their immune surveillance function. Nevertheless, the molecular mechanisms by which this occurs remain unclear. This process is likely to be highly regulated. We propose that the cytosolic delivery of macromolecular extracellular “danger” signals to cytosolic sensors is regulated at three key stages. First, entry into the host cell occurs *via* receptor-mediated endocytosis. Dedicated receptors on the surface of phagocytes either bind directly to the “danger” signal, or indirectly *via* protein bridges or chaperones such as HMGB1 and LL37. This serves to regulate the type of cargo being delivered to the cytosol, but also, by virtue of selective expression of the receptors, serves to target the “danger” signals to sentinel cells such as macrophages and dendritic cells. Second, receptor signaling results in the destabilization of the endocytic organelle membrane by mechanisms such as, but not limited to, NADPH dependent-ROS production. Both DNGR-1 and RAGE induce robust NADPH oxidase-dependent ROS production which may directly destabilize the limiting membranes of endocytic organelles or may in some way mark the endocytic organelles for destabilization by other protein effectors. Receptor-engagement provides an added layer of regulation resulting in the selective and inducible destabilization of endocytic organelles carrying “danger” signals. Finally, membrane destabilization results in the delivery of the “danger” signal to the cytosol for detection by cytosolic sensors. Although currently unknown, we anticipate that such membrane destabilization is transient and is likely repaired by membrane repair machinery such as the ESCRT pathway. Similarly, damaged organelles may also be replenished *via* organelle biogenesis (85–87). With already defined roles in tumor immunity, sepsis and antigen presentation, the detection of extracellular “danger” signals by cytosolic sensors will continue to emerge as a fundamental mechanism by which sentinel cells such as macrophages and dendritic cells achieve their roles in immunity and homeostasis. Understanding the mechanisms driving these processes is therefore of critical importance.

## Author Contributions

GG and JC wrote the manuscript. All authors contributed to the article and approved the submitted version.

## Conflict of Interest

The authors declare that the research was conducted in the absence of any commercial or financial relationships that could be construed as a potential conflict of interest.

## Publisher’s Note

All claims expressed in this article are solely those of the authors and do not necessarily represent those of their affiliated organizations, or those of the publisher, the editors and the reviewers. Any product that may be evaluated in this article, or claim that may be made by its manufacturer, is not guaranteed or endorsed by the publisher.

## References

[1] Han CZ Ravichandran KS. Metabolic Connections During Apoptotic Cell Engulfment. Cell (2011) 147:1442–5. doi: 10.1016/j.cell.2011.12.006 PMC3254670 22196723

[2] Freeman SA Grinstein S. Resolution of Macropinosomes, Phagosomes and Autolysosomes: Osmotically Driven Shrinkage Enables Tubulation and Vesiculation. Traffic (2018) 19:965–74. doi: 10.1111/tra.12614 30159984

[3] Freeman SA Uderhardt S Saric A Collins RF Buckley CM Mylvaganam S. Lipid-Gated Monovalent Ion Fluxes Regulate Endocytic Traffic and Support Immune Surveillance. Science (2020) 367:301–5. doi: 10.1126/science.aaw9544 PMC8118712 31806695

[4] Levin R Grinstein S Canton J. The Life Cycle of Phagosomes: Formation, Maturation, and Resolution. Immunol Rev (2016) 273:156–79. doi: 10.1111/imr.12439 27558334

[5] Blum JS Wearsch PA Cresswell P. Pathways of Antigen Processing. Annu Rev Immunol (2013) 31:443–73. doi: 10.1146/annurev-immunol-032712-095910 PMC4026165 23298205

[6] Grotzke JE Sengupta D Lu Q Cresswell P. The Ongoing Saga of the Mechanism(s) of MHC Class I-Restricted Cross-Presentation. Curr Opin Immunol (2017) 46:89–96. doi: 10.1016/j.coi.2017.03.015 28528219 PMC5554740

[7] Theisen D Murphy K. The Role of Cdc1s *In Vivo*: CD8 T Cell Priming Through Cross-Presentation. F1000Res (2017) 6:98. doi: 10.12688/f1000research.9997.1 28184299 PMC5288679

[8] Murphy TL Murphy KM. Dendritic Cells in Cancer Immunology. Cell Mol Immunol (2022) 19:3–13. doi: 10.1038/s41423-021-00741-5 34480145 PMC8752832

[9] Theisen DJ. WDFY4 is Required for Cross-Presentation in Response to Viral and Tumor Antigens. Science (2018) 362:694–9. doi: 10.1126/science.aat5030 PMC6655551 30409884

[10] Hildner K Edelson BT Purtha WE Diamond M Matsushita H Kohyama M. Batf3 Deficiency Reveals a Critical Role for CD8α+ Dendritic Cells in Cytotoxic T Cell Immunity. Science (2008) 322:1097–100. doi: 10.1126/science.1164206 PMC2756611 19008445

[11] Canton J Blees H Henry CM Buck MD Schulz O Rogers NC. The Receptor DNGR-1 Signals for Phagosomal Rupture to Promote Cross-Presentation of Dead-Cell-Associated Antigens. Nat Immunol (2021) 22:140–53. doi: 10.1038/s41590-020-00824-x PMC7116638 33349708

[12] ancho D Joffre OP Keller AM Rogers NC Martínez D Hernanz-Falcón P. Identification of a Dendritic Cell Receptor That Couples Sensing of Necrosis to Immunity. Nature (2009) 458:899–903. doi: 10.1038/nature07750 19219027 PMC2671489

[13] Giampazolias E Schulz O Lim KHJ Rogers NC Chakravarty P Srinivasan N. Secreted Gelsolin Inhibits DNGR-1-Dependent Cross-Presentation and Cancer Immunity. Cell (2021) 184:4016–31.e22. doi: 10.1016/j.cell.2021.05.021 PMC8320529 34081922

[14] de Mingo Pulido Á Hänggi K Celias DP Gardner A Li J Batista-Bittencourt B. The Inhibitory Receptor TIM-3 Limits Activation of the cGAS-STING Pathway in Intra-Tumoral Dendritic Cells by Suppressing Extracellular DNA Uptake. Immunity (2021) 54:1154–67.e7. doi: 10.1016/j.immuni.2021.04.019 33979578 PMC8192496

[15] Ahn J Xia T Rabasa Capote A Betancourt D Barber GN. Extrinsic Phagocyte-Dependent STING Signaling Dictates the Immunogenicity of Dying Cells. Cancer Cell (2018) 33:862–73.e5. doi: 10.1016/j.ccell.2018.03.027 29706455 PMC6177226

[16] Woo S-R Fuertes MB Corrales L Spranger S Furdyna MJ Leung MYK. STING-Dependent Cytosolic DNA Sensing Mediates Innate Immune Recognition of Immunogenic Tumors. Immunity (2014) 41:830–42. doi: 10.1016/j.immuni.2014.10.017 PMC4384884 25517615

[17] Vanaja SK Russo AJ Behl B Banerjee I Yankova M Deshmukh SD. Bacterial Outer Membrane Vesicles Mediate Cytosolic Localization of LPS and Caspase-11 Activation. Cell (2016) 165:1106–19. doi: 10.1016/j.cell.2016.04.015 PMC4874922 27156449

[18] Vasudevan SO Russo AJ Kumari P Vanaja SK. & Rathinam, V. A. A TLR4-Independent Critical Role for CD14 in Intracellular LPS Sensing. Cell Rep (2022) 39:110755. doi: 10.1016/j.celrep.2022.110755 35508125 PMC9376664

[19] Deng M Tang Y Li W Wang X Zhang R Zhang X. The Endotoxin Delivery Protein HMGB1 Mediates Caspase-11-Dependent Lethality in Sepsis. Immunity (2018) 49:740–53.e7. doi: 10.1016/j.immuni.2018.08.016 30314759 PMC6300139

[20] Tang Y Wang X Li Z He Z Yang X Cheng X. Heparin Prevents Caspase-11-Dependent Septic Lethality Independent of Anticoagulant Properties. Immunity (2021) 54:454–67.e6. doi: 10.1016/j.immuni.2021.01.007 33561388

[21] Huang AYC Golumbek P Ahmadzadeh M Jaffee E Pardoll D Levitsky H. Role of Bone Marrow-Derived Cells in Presenting MHC Class I-Restricted Tumor Antigens. Science (1994) 264:961–5. doi: 10.1126/science.7513904 7513904

[22] Sigal LJ Crotty S Andino R Rock KL. Cytotoxic T-Cell Immunity to Virus-Infected non-Haematopoietic Cells Requires Presentation of Exogenous Antigen. Nature (1999) 398:77–80. doi: 10.1038/18038 10078533

[23] den Haan JMM Bevan MJ. Antigen Presentation to CD8+ T Cells: Cross-Priming in Infectious Diseases. Curr Opin Immunol (2001) 13:437–41. doi: 10.1016/S0952-7915(00)00238-7 11498299

[24] Harding CV Song R. Phagocytic Processing of Exogenous Particulate Antigens by Macrophages for Presentation by Class I MHC Molecules. J Immunol (1994) 153:4925–33. 7963555

[25] Song R Harding CV. Roles of Proteasomes, Transporter for Antigen Presentation (TAP), and Beta 2-Microglobulin in the Processing of Bacterial or Particulate Antigens *via* an Alternate Class I MHC Processing Pathway. J Immunol (1996) 156:4182–90. 8666786

[26] Pfeifer JD Wick MJ Roberts RL Findlay K Normark SJ Harding CV. Phagocytic Processing of Bacterial Antigens for Class I MHC Presentation to T Cells. Nature (1993) 361:359–62. doi: 10.1038/361359a0 7678924

[27] Kovacsovics-Bankowski M. & Rock, K. L. A Phagosome-to-Cytosol Pathway for Exogenous Antigens Presented on MHC Class I Molecules. Science (1995) 267:243–6. doi: 10.1126/science.7809629 7809629

[28] Reis e Sousa C Germain RN. Major Histocompatibility Complex Class I Presentation of Peptides Derived From Soluble Exogenous Antigen by a Subset of Cells Engaged in Phagocytosis. J Exp Med (1995) 182:841–51. doi: 10.1084/jem.182.3.841 PMC2192173 7650490

[29] Oh YK Harding CV Swanson JA. The Efficiency of Antigen Delivery From Macrophage Phagosomes Into Cytoplasm for MHC Class I-Restricted Antigen Presentation. Vaccine (1997) 15:511–8. doi: 10.1016/S0264-410X(97)00221-1 9160518

[30] Norbury CC Hewlett LJ Prescott AR Shastri N Watts C. Class I MHC Presentation of Exogenous Soluble Antigen *via* Macropinocytosis in Bone Marrow Macrophages. Immunity (1995) 3:783–91. doi: 10.1016/1074-7613(95)90067-5 8777723

[31] Cebrian I Visentin G Blanchard N Jouve M Bobard A Moita C. Sec22b Regulates Phagosomal Maturation and Antigen Crosspresentation by Dendritic Cells. Cell (2011) 147:1355–68. doi: 10.1016/j.cell.2011.11.021 22153078

[32] Lin ML Zhan Y Proietto AI Prato S Wu L Heath WR. Selective Suicide of Cross-Presenting CD8+ Dendritic Cells by Cytochrome C Injection Shows Functional Heterogeneity Within This Subset. Proc Natl Acad Sci U.S.A. (2008) 105:3029–34. doi: 10.1073/pnas.0712394105 PMC2268579 18272486

[33] Giodini A Cresswell P. Hsp90-Mediated Cytosolic Refolding of Exogenous Proteins Internalized by Dendritic Cells. EMBO J (2008) 27:201–11. doi: 10.1038/sj.emboj.7601941 PMC2104710 18046456

[34] Ackerman AL Kyritsis C Tampé R Cresswell P. Early Phagosomes in Dendritic Cells Form a Cellular Compartment Sufficient for Cross Presentation of Exogenous Antigens. Proc Natl Acad Sci U.S.A. (2003) 100:12889–94. doi: 10.1073/pnas.1735556100 PMC240714 14561893

[35] Palmowski MJ Gileadi U Salio M Gallimore A Millrain M James E. Role of Immunoproteasomes in Cross-Presentation. J Immunol (2006) 177:983–90. doi: 10.4049/jimmunol.177.2.983 16818754

[36] Enders M Franken L Philipp MS Kessler N Baumgart AK Eichler M. Splenic Red Pulp Macrophages Cross-Prime Early Effector CTL That Provide Rapid Defense Against Viral Infections. J Immunol (2020) 204:87–100. doi: 10.4049/jimmunol.1900021 31776205

[37] Garin J Diez R Kieffer S Dermine JF Duclos S Gagnon E. The Phagosome Proteome. J Cell Biol (2001) 152:165–80. doi: 10.1083/jcb.152.1.165 PMC2193653 11149929

[38] Alloatti A Rookhuizen DC Joannas L Carpier JM Iborra S Magalhaes JG. Critical Role for Sec22b-Dependent Antigen Cross-Presentation in Antitumor Immunity. J Exp Med (2017) 214:2231–41. doi: 10.1084/jem.20170229 PMC5551575 28663435

[39] Zehner M Marschall AL Bos E Schloetel JG Kreer C Fehrenschild D. The Translocon Protein Sec61 Mediates Antigen Transport From Endosomes in the Cytosol for Cross-Presentation to CD8+ T Cells. Immunity (2015) 42:850–63. doi: 10.1016/j.immuni.2015.04.008 25979419

[40] Grotzke JE Cresswell P. Are ERAD Components Involved in Cross-Presentation? Mol Immunol (2015) 68:112–5. doi: 10.1016/j.molimm.2015.05.002 26005101

[41] Koopmann J-O Albring J Hüter E Bulbuc N Spee P Neefjes J. Export of Antigenic Peptides From the Endoplasmic Reticulum Intersects With Retrograde Protein Translocation Through the Sec61p Channel. Immunity (2000) 13:117–27. doi: 10.1016/S1074-7613(00)00013-3 10933400

[42] Ackerman AL Giodini A. & Cresswell, P. A Role for the Endoplasmic Reticulum Protein Retrotranslocation Machinery During Crosspresentation by Dendritic Cells. Immunity (2006) 25:607–17. doi: 10.1016/j.immuni.2006.08.017 17027300

[43] Grotzke JE Kozik P Morel JD Impens F Pietrosemoli N Cresswell P. Sec61 Blockade by Mycolactone Inhibits Antigen Cross-Presentation Independently of Endosome-to-Cytosol Export. Proc Natl Acad Sci (2017) 114:E5910–9. doi: 10.1073/pnas.1705242114 PMC5530691 28679634

[44] Leibowitz ME Johkson MC. Relation of Lipid Peroxidation to Loss of Cations Trapped in Liposomes. J Lipid Res (1971) 12:662–70. doi: 10.1016/S0022-2275(20)39453-0 5124531

[45] Stark G. The Effect of Ionizing Radiation on Lipid Membranes. Biochim Biophys Acta (BBA) - Rev Biomembranes (1991) 1071:103–22. doi: 10.1016/0304-4157(91)90020-W 1854791

[46] Wong-ekkabut J Xu Z Triampo W Tang IM Tieleman DP Monticelli L. Effect of Lipid Peroxidation on the Properties of Lipid Bilayers: A Molecular Dynamics Study. Biophys J (2007) 93:4225–36. doi: 10.1529/biophysj.107.112565 PMC2098729 17766354

[47] Dingjan I Verboogen DR Paardekooper LM Revelo NH Sittig SP Visser LJ. Lipid Peroxidation Causes Endosomal Antigen Release for Cross-Presentation. Sci Rep (2016) 6:22064. doi: 10.1038/srep22064 26907999 PMC4764948

[48] Dingjan I Paardekooper LM Verboogen DRJ von Mollard GF Ter Beest M van den Bogaart G. VAMP8-Mediated NOX2 Recruitment to Endosomes is Necessary for Antigen Release. Eur J Cell Biol (2017) 96:705–14. doi: 10.1016/j.ejcb.2017.06.007 PMC5641923 28688576

[49] Nalle SC Barreira da Silva R Zhang H Decker M Chalouni C Xu M. Aquaporin-3 Regulates Endosome-to-Cytosol Transfer *via* Lipid Peroxidation for Cross Presentation. PloS One (2020) 15:e0238484. doi: 10.1371/journal.pone.0238484 33232321 PMC7685505

[50] Gros M Amigorena S. Regulation of Antigen Export to the Cytosol During Cross-Presentation. Front Immunol (2019) 10:. doi: 10.3389/fimmu.2019.00041 PMC6360170 30745902

[51] Hatinguais R Brown GD. Cross-Presentation is Getting DNGRous. Nat Immunol (2021) 22:108–10. doi: 10.1038/s41590-020-00831-y 33398180

[52] Schulz O Hanč P Böttcher JP Hoogeboom R Diebold SS Tolar P. Myosin II Synergizes With F-Actin to Promote DNGR-1-Dependent Cross-Presentation of Dead Cell-Associated Antigens. Cell Rep (2018) 24:419–28. doi: 10.1016/j.celrep.2018.06.038 PMC6057488 29996102

[53] Childs E Henry CM Canton J Reis e Sousa C. Maintenance and Loss of Endocytic Organelle Integrity: Mechanisms and Implications for Antigen Cross-Presentation. Open Biol (2021) 11:210194. doi: 10.1098/rsob.210194 34753318 PMC8580422

[54] Radulovic M Schink KO Wenzel EM Nähse V Bongiovanni A Lafont F. ESCRT-Mediated Lysosome Repair Precedes Lysophagy and Promotes Cell Survival. EMBO J (2018) 37:e99753. doi: 10.15252/embj.201899753 30314966 PMC6213280

[55] Skowyra ML Schlesinger PH Naismith TV Hanson PI. Triggered Recruitment of ESCRT Machinery Promotes Endolysosomal Repair. Sci 360 Eaar5078 (2018) 360:eaar5078. doi: 10.1126/science.aar5078 PMC6195421 29622626

[56] Lande R Gregorio J Facchinetti V Chatterjee B Wang YH Homey B. Plasmacytoid Dendritic Cells Sense Self-DNA Coupled With Antimicrobial Peptide. Nature (2007) 449:564–9. doi: 10.1038/nature06116 17873860

[57] Chamilos G Gregorio J Meller S Lande R Kontoyiannis DP Modlin RL. Cytosolic Sensing of Extracellular Self-DNA Transported Into Monocytes by the Antimicrobial Peptide LL37. Blood (2012) 120:3699–707. doi: 10.1182/blood-2012-01-401364 PMC3488884 22927244

[58] Yanai H Ban T Wang Z Choi MK Kawamura T Negishi H. HMGB Proteins Function as Universal Sentinels for Nucleic-Acid-Mediated Innate Immune Responses. Nature (2009) 462:99–103. doi: 10.1038/nature08512 19890330

[59] Li X Yue Y Zhu Y Xiong S. Extracellular, But Not Intracellular HMGB1, Facilitates Self-DNA Induced Macrophage Activation *via* Promoting DNA Accumulation in Endosomes and Contributes to the Pathogenesis of Lupus Nephritis. Mol Immunol (2015) 65:177–88. doi: 10.1016/j.molimm.2015.01.023 25660970

[60] Lu J Yue Y Xiong S. Extracellular HMGB1 Augments Macrophage Inflammation by Facilitating the Endosomal Accumulation of ALD-DNA *via* TLR2/4-Mediated Endocytosis. Biochim Biophys Acta (BBA) - Mol Basis Dis (2021) 1867:166184. doi: 10.1016/j.bbadis.2021.166184 34087422

[61] Lande R Ganguly D Facchinetti V Frasca L Conrad C Gregorio J. Neutrophils Activate Plasmacytoid Dendritic Cells by Releasing Self-DNA–Peptide Complexes in Systemic Lupus Erythematosus. Sci Trans Med (2011) 3:73ra19–9. doi: 10.1126/scitranslmed.3001180 PMC3399524 21389263

[62] Apel F Andreeva L Knackstedt LS Streeck R Frese CK Goosmann C. The Cytosolic DNA Sensor cGAS Recognizes Neutrophil Extracellular Traps. Sci Signaling 14 Eaax7942 (2021) 14:eaax7942. doi: 10.1126/scisignal.aax7942 33688080

[63] GUO Z PENG H KANG J SUN D. Cell-Penetrating Peptides: Possible Transduction Mechanisms and Therapeutic Applications. BioMed Rep (2016) 4:528–34. doi: 10.3892/br.2016.639 PMC4840506 27123243

[64] Ruseska I Zimmer A. Internalization Mechanisms of Cell-Penetrating Peptides. Beilstein J Nanotechnol (2020) 11:101–23. doi: 10.3762/bjnano.11.10 PMC6964662 31976201

[65] Brubaker SW Bonham KS Zanoni I Kagan JC. Innate Immune Pattern Recognition: A Cell Biological Perspective. Annu Rev Immunol (2015) 33:257–90. doi: 10.1146/annurev-immunol-032414-112240 PMC5146691 25581309

[66] Ragland SA Kagan JC. Cytosolic Detection of Phagosomal Bacteria-Mechanisms Underlying PAMP Exodus From the Phagosome Into the Cytosol. Mol Microbiol (2021) 116:1420–32. doi: 10.1111/mmi.14841 PMC8688326 34738270

[67] Trindade BC Chen GY. NOD1 and NOD2 in Inflammatory and Infectious Diseases. Immunol Rev (2020) 297:139–61. doi: 10.1111/imr.12902 PMC8928416 32677123

[68] Lee J Tattoli I Wojtal KA Vavricka SR Philpott DJ Girardin SE. pH-Dependent Internalization of Muramyl Peptides From Early Endosomes Enables Nod1 and Nod2 Signaling. J Biol Chem (2009) 284:23818–29. doi: 10.1074/jbc.M109.033670 PMC2749154 19570976

[69] Nakamura N Lill JR Phung Q Jiang Z Bakalarski C de Mazière A. Endosomes are Specialized Platforms for Bacterial Sensing and NOD2 Signalling. Nature (2014) 509:240–4. doi: 10.1038/nature13133 24695226

[70] Hu Y Song F Jiang H Nuñez G Smith DE. SLC15A2 and SLC15A4 Mediate the Transport of Bacterially Derived Di/Tripeptides To Enhance the Nucleotide-Binding Oligomerization Domain-Dependent Immune Response in Mouse Bone Marrow-Derived Macrophages. J Immunol (2018) 201:652–62. doi: 10.4049/jimmunol.1800210 PMC6039277 29784761

[71] Canton J Schlam D Breuer C Gütschow M Glogauer M Grinstein S. Calcium-Sensing Receptors Signal Constitutive Macropinocytosis and Facilitate the Uptake of NOD2 Ligands in Macrophages. Nat Commun (2016) 7:1–12. doi: 10.1038/ncomms11284 PMC4823870 27050483

[72] He W Wan H Hu L Chen P Wang X Huang Z. Gasdermin D is an Executor of Pyroptosis and Required for Interleukin-1β Secretion. Cell Res (2015) 25:1285–98. doi: 10.1038/cr.2015.139 PMC4670995 26611636

[73] Cheng KT Xiong S Ye Z Hong Z Di A Tsang KM. Caspase-11–Mediated Endothelial Pyroptosis Underlies Endotoxemia-Induced Lung Injury. J Clin Invest (2017) 127:4124–35. doi: 10.1172/JCI94495 PMC5663346 28990935

[74] Piras S Furfaro AL Domenicotti C Traverso N Marinari UM Pronzato MA. RAGE Expression and ROS Generation in Neurons: Differentiation Versus Damage. Oxid Med Cell Longev (2016) 2016:9348651. doi: 10.1155/2016/9348651 27313835 PMC4897723

[75] Yao D Brownlee M. Hyperglycemia-Induced Reactive Oxygen Species Increase Expression of the Receptor for Advanced Glycation End Products (RAGE) and RAGE Ligands. Diabetes (2010) 59:249–55. doi: 10.2337/db09-0801 PMC2797929 19833897

[76] Wautier MP Chappey O Corda S Stern DM Schmidt AM Wautier JL. Activation of NADPH Oxidase by AGE Links Oxidant Stress to Altered Gene Expression *via* RAGE. Am J Physiol Endocrinol Metab (2001) 280:E685–94. doi: 10.1152/ajpendo.2001.280.5.E685 11287350

[77] Meunier E Dick MS Dreier RF Schürmann N Kenzelmann Broz D Warming S. Caspase-11 Activation Requires Lysis of Pathogen-Containing Vacuoles by IFN-Induced GTPases. Nature (2014) 509:366–70. doi: 10.1038/nature13157 24739961

[78] Finethy R Luoma S Orench-Rivera N Feeley EM Haldar AK Yamamoto M. Inflammasome Activation by Bacterial Outer Membrane Vesicles Requires Guanylate Binding Proteins. mBio (2017) 8:e01188–17. doi: 10.1128/mBio.01188-17 PMC5626967 28974614

[79] Fisch D Bando H Clough B Hornung V Yamamoto M Shenoy AR. Human GBP1 is a Microbe-Specific Gatekeeper of Macrophage Apoptosis and Pyroptosis. EMBO J (2019) 38:e100926. doi: 10.15252/embj.2018100926 31268602 PMC6600649

[80] Wandel MP Kim BH Park ES Boyle KB Nayak K Lagrange B. Guanylate-Binding Proteins Convert Cytosolic Bacteria Into Caspase-4 Signaling Platforms. Nat Immunol (2020) 21:880–91. doi: 10.1038/s41590-020-0697-2 PMC7381384 32541830

[81] Santos JC Boucher D Schneider LK Demarco B Dilucca M Shkarina K. Human GBP1 Binds LPS to Initiate Assembly of a Caspase-4 Activating Platform on Cytosolic Bacteria. Nat Commun (2020) 11:3276. doi: 10.1038/s41467-020-16889-z 32581219 PMC7314798

[82] Santos JC Broz P. Sensing of Invading Pathogens by GBPs: At the Crossroads Between Cell-Autonomous and Innate Immunity. J Leuk Biol (2018) 104:729–35. doi: 10.1002/JLB.4MR0118-038R 30020539

[83] Yang J Hwang I Lee E Shin SJ Lee EJ Rhee JH. Bacterial Outer Membrane Vesicle-Mediated Cytosolic Delivery of Flagellin Triggers Host NLRC4 Canonical Inflammasome Signaling. Front Immunol (2020) 11. doi: 10.3389/fimmu.2020.581165 PMC7708323 33312172

[84] Tenthorey JL Haloupek N López-Blanco JR Grob P Adamson E Hartenian E. The Structural Basis of Flagellin Detection by NAIP5: A Strategy to Limit Pathogen Immune Evasion. Science (2017) 358:888–93. doi: 10.1126/science.aao1140 PMC5842810 29146805

[85] Saftig P Klumperman J. Lysosome Biogenesis and Lysosomal Membrane Proteins: Trafficking Meets Function. Nat Rev Mol Cell Biol (2009) 10:623–35. doi: 10.1038/nrm2745 19672277

[86] Idone V Tam C Andrews NW. Two-Way Traffic on the Road to Plasma Membrane Repair. Trends Cell Biol (2008) 18:552–9. doi: 10.1016/j.tcb.2008.09.001 PMC2593466 18848451

[87] Roy D Liston DR Idone VJ Di A Nelson DJ Pujol C. A Process for Controlling Intracellular Bacterial Infections Induced by Membrane Injury. Science (2004) 304:1515–8. doi: 10.1126/science.1098371 15178804

